# Cardioembolic Breakthrough Stroke: When Anticoagulation Fails, Is Left Atrial Appendage Closure the Next Step?

**DOI:** 10.1111/ene.70387

**Published:** 2025-10-15

**Authors:** Lina Palaiodimou, Georgios Tsivgoulis

**Affiliations:** ^1^ Second Department of Neurology “Attikon” University Hospital, School of Medicine, National and Kapodistrian University of Athens Athens Greece

**Keywords:** anticoagulation, atrial fibrillation, breakthrough stroke, left atrial appendage occlusion

Atrial fibrillation (AF) is the most common sustained arrhythmia and is associated with up to a fivefold increased risk of ischemic stroke. Moreover, AF has been strongly linked to early recurrence after ischemic stroke, making secondary prevention both urgent and complex. Oral anticoagulation (OAC) remains the cornerstone of prevention, reducing thromboembolic risk substantially in both primary and secondary settings. However, despite meticulous adherence and adequate dosing, more than 20% of patients in real‐world cohorts experience acute ischemic stroke or transient ischemic attack while on anticoagulation [1]. Notably, shifting from one anticoagulant to another after such an event has not been shown to reduce recurrence risk [2]. This sobering reality underscores the need for better strategies in secondary prevention.

Against this backdrop, Galea and colleagues report an international observational study on percutaneous left atrial appendage closure (LAAC) in patients with AF who suffered a cardioembolic breakthrough stroke despite adequate OAC [3]. From four European centers, they identified 95 rigorously adjudicated cases: patients who had an ischemic stroke clearly attributable to cardioembolism and who were adequately anticoagulated at the time of their event. Procedures were performed at a median of 4 months after the index stroke. At 2 years' follow‐up, recurrent ischemic stroke occurred in just 4% of patients. Device‐related thrombus was infrequent (2%), while peri‐device leaks were documented in nearly one in ten patients, though only a minority were clinically significant. Procedural complications occurred in 1%. Importantly, these procedural adverse events were expected and are in line with prior large LAAC trials, underscoring both the feasibility and the known limitations of this approach [4].

These findings speak directly to a population at the margins of our current evidence base. Guidelines today reserve LAAC primarily for patients with AF who have contraindications to anticoagulation—for example, those with high bleeding risk, cerebral amyloid angiopathy, or renal failure. The present study raises the possibility of a different role: LAAC as an adjunct to anticoagulation in patients with recurrent events despite adequate OAC. Such an approach may offer additive protection, by physically eliminating the left atrial appendage—the predominant nidus of thrombus formation. If validated, this strategy could fill a critical therapeutic gap: patients in whom the “best available” medical therapy is demonstrably insufficient.

Still, enthusiasm must be tempered by important limitations. This was a retrospective analysis without a control arm. And while recurrent stroke was reduced, antithrombotic therapy after LAAC was heterogeneous, with most patients remaining on OAC. This raises the following question: is the benefit attributable to the device, the medication, or their combination? Another nuance is mechanistic. LAAC does not address atrial cardiomyopathy, atrial stasis outside the appendage, or non‐cardioembolic pathways. Thus, it cannot be expected to abolish all recurrent risk. Indeed, some events observed in this study were likely due to small vessel disease or periprocedural factors unrelated to the LAA. This underlines the importance of careful etiologic adjudication and multimodal secondary prevention.

The findings of Galea and colleagues should also be interpreted in light of prior observational evidence [5, 6, 7, 8, 9, 10]. Across prior cohorts, recurrence rates of ischemic stroke ranged from as low as 1.9 to as high as 7.7 strokes per 100 patient‐years. Importantly, many of these studies did not systematically exclude patients with competing stroke etiologies or inadequate anticoagulation, factors that may have diluted the apparent benefit of LAAC. By contrast, Galea et al. restricted their analysis to rigorously adjudicated cardioembolic breakthrough strokes under adequate OAC, providing a clearer signal of efficacy. When these seven studies were pooled in a random‐effects meta‐analysis (Figure 1), encompassing 1251 patients, the overall recurrent ischemic stroke rate was estimated at 4% (95% CI, 3%–5%), with no evidence of statistical heterogeneity (*I* ^2^ = 0%). This consistency across cohorts reinforces the notion that LAAC is both feasible and protective in this high‐risk group.

**FIGURE 1:**
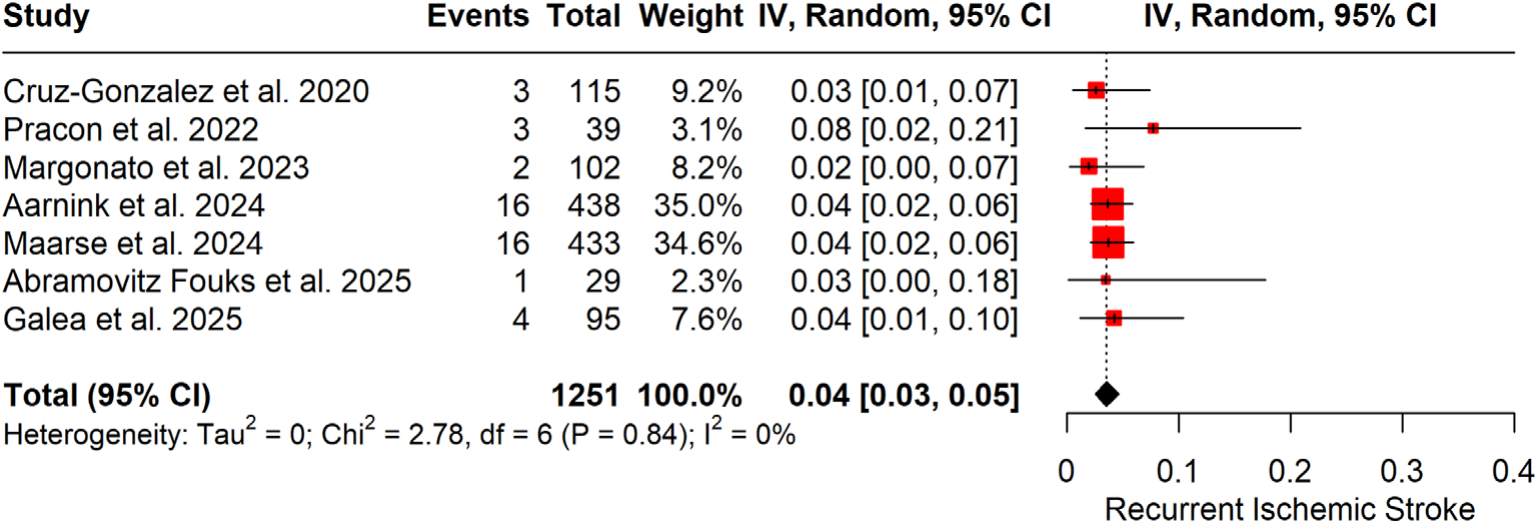
Forest plot presenting the pooled proportion of recurrent ischemic stroke among patients with breakthrough stroke undergoing left atrium appendage closure in addition to antithrombotic treatment. Pooled proportions with 95% confidence intervals were estimated using the variance‐stabilizing double arcsine transformation. Statistical analysis was performed in R (version 4.4.2) with the meta package.

Randomized‐controlled clinical trials will be decisive. Two are now underway. The ELAPSE trial (NCT05976685) is enrolling patients with AF and cardioembolic breakthrough stroke, randomizing them to OAC alone or OAC plus LAAC within 3 months of their index event. LAAOS‐4 (NCT05963698) is enrolling a broader AF population with CHA_2_DS_2_‐VASc ≥ 4, comparing OAC alone with OAC plus LAAC. Together, these studies will provide the first high‐level evidence to clarify whether LAAC truly confers additive benefit in this population. Results, however, are years away.

For clinicians, what should be done today? Anticoagulation remains foundational, and LAAC cannot replace it in most patients. But for those who suffer ischemic stroke despite adequate therapy, and particularly for those at high recurrence risk, LAAC may reasonably be considered in specialized centers with procedural expertise. The decision must be individualized, grounded in multidisciplinary discussion, and framed as an adjunctive—not alternative—strategy pending definitive trial results.

## Author Contributions

**Lina Palaiodimou:** writing – original draft, investigation, methodology, visualization, formal analysis, data curation. **Georgios Tsivgoulis:** conceptualization, investigation, writing – original draft, methodology, validation, visualization, formal analysis, data curation, supervision.

## Conflicts of Interest

The authors declare no conflicts of interest.

## Data Availability

The data that support the findings of this study are available from the corresponding author upon reasonable request.
